# Rethinking wellbeing measurement: Learning from a scale development study with adolescents living with HIV in Zimbabwe

**DOI:** 10.1371/journal.pgph.0006845

**Published:** 2026-09-30

**Authors:** Webster Mavhu, Jermaine M. Dambi, Victoria Simms, Faith Martin, Joni Lariat, Rufaro Mbundure, Leviticus Makoni, Laura Kafata, Carol Wogrin, Vivian Chitiyo, Abigail Mutsinze, Frances M. Cowan, Nicola Willis, Sarah Bernays

**Affiliations:** 1 Sexual and Reproductive Health Department, Centre for Sexual Health and HIV/AIDS Research (CeSHHAR), Harare, Zimbabwe; 2 Department of International Public Health, Liverpool School of Tropical Medicine, Liverpool, United Kingdom; 3 Rehabilitation Sciences Unit, Faculty of Medicine and Health Sciences, University of Zimbabwe, Harare, Zimbabwe; 4 Friendship Bench, Harare, Zimbabwe; 5 MRC International Statistics and Epidemiology Group, London School of Hygiene & Tropical Medicine, London, United Kingdom; 6 Department of Psychology, University of Bath, Somerset, United Kingdom; 7 Sydney School of Public Health, University of Sydney, Camperdown, NSW, Australia; 8 Zvandiri, Harare, Zimbabwe; 9 Global Health and Development, London School of Hygiene & Tropical Medicine, London, United Kingdom; University of the Witwatersrand Johannesburg Faculty of Health Sciences, SOUTH AFRICA

## Abstract

Adolescents living with HIV (ALHIV) in Eastern and Southern Africa experience challenges around medication adherence, mental health and wellbeing. Although interventions such as Zvandiri aim to strengthen mental health and wellbeing to impact HIV treatment outcomes, available measures often focus on negative outcomes or rely on positive psychology instruments developed in high-income settings. These tools may not adequately capture culturally embedded and relational dimensions of wellbeing. We describe the development of the Zvandiri Character Strength (ZCS) scale and draw methodological lessons for culturally grounded wellbeing measurement. The multi-stage scale-development process (2022–2025) comprised a scoping and systematic review, qualitative discussions, expert panel input, construct and item prioritisation, cognitive interviews, iterative item reduction, and exploratory factor and Rasch analyses. We reduced an initial item bank to a 53-item candidate scale, then to the ZCS 38 (38 items) and finally, the ZCS 25 (25 items), based on quantitative analyses and participant feedback. We subsequently assessed the ZCS 25 using retrospective pretest, post-test and follow-up administration. The review identified conceptually overlapping positive psychological constructs, many of which are measured using instruments developed in high-income settings and not adequately validated for ALHIV in Africa. Qualitative work highlighted connectedness, hope, self-acceptance, self-efficacy, purpose, and flourishing as key. Discussions also highlighted that wellbeing was relational, contextually situated, and often co-occurring with distress. Quantitative testing showed highly positive responses, disordered thresholds, ceiling effects, weak internal structure, and fluctuating retrospective ratings. Participant feedback suggested that positive responses likely reflected affirmations, and were partly linked to social desirability. In conclusion, the ZCS scale did not support a single interpretable total score, but its development offers important methodological lessons. Future measurement should begin with clearer construct definition, response-process validation, domain-level testing and explicit decisions about whether the measure aims to capture lived wellbeing, domain-specific positive outcomes or intervention-relevant change.

## Introduction

Globally, adolescents living with HIV (ALHIV) have the highest rates of attrition from HIV treatment and care of any age group, resulting in higher rates of treatment failure, morbidity and mortality compared to children and adults [1,2]. In Eastern and Southern Africa, which is home to 84% of ALHIV globally, adolescents are the only population with increasing mortality rates [3,4]. Elevated mortality rates are exacerbated by the significant prevalence of common mental health conditions [5]. Poor mental health is important in its own right, but also because it impacts adherence to HIV medication. For example, depression and anxiety significantly hinder treatment adherence, which in turn results in higher mortality [6].

Zvandiri (meaning ‘As I am’ in Shona, www.zvandiri.org) is a theoretically-grounded, multi-component differentiated service delivery model for children, adolescents and young adults living with HIV (CAYALHIV) [2,7,8]. Zvandiri links CAYALHIV to Community Adolescent Treatment Supporters (CATS); peer counsellors who are also living with HIV. These trained and mentored supporters help improve CAYALHIV’s health, wellbeing, and engagement with HIV treatment and care [2,9–11]. In a cluster-randomised trial of the Zvandiri model, ALHIV receiving the Zvandiri intervention had 42% lower prevalence of virological failure or death after 2 years compared to ALHIV receiving standard care alone (95% CI: 0.36-0.94) [2,12,13]. A subsequent trial assessed the effect of training CATS in problem-solving therapy and found a five-fold reduction in the odds of common mental disorder symptoms [14].

Our past research suggests that improved mental health and wellbeing enable medication adherence [15,16]. Nevertheless, a gap persists in the comprehensive evaluation of wellbeing, since most of the research to date prioritises negative indicators such as depression and anxiety [17,18]. Of note, reliance on measuring negative outcomes undermines our capacity to identify and quantify the development of protective attributes, as proactive prevention.

Positive constructs have not been wholly absent from ALHIV research. Our review identified a range of positive psychological outcomes already used in this field, including resilience, self-esteem, self-efficacy, self-worth, coping, flourishing, and positive outlook [17]. The problem is that this literature is conceptually fragmented, with overlapping constructs often measured with instruments developed in high-income settings that are not adequately adapted or validated for ALHIV in Eastern and Southern Africa [19]. The task, therefore, is to develop culturally appropriate, meaningful and psychometrically defensible approaches to assessing the positive outcomes and mechanisms through which interventions may improve young people’s lives.

ALHIV interventions may plausibly affect outcomes such as belonging, self-worth, treatment confidence, future orientation, resilience and relational safety, even where symptom-based or virological outcomes do not fully capture change. Measuring these domains is therefore important, but it requires clarity about which construct is being assessed, why it matters to the intervention theory of change, and whether the measure is valid for the language, context and social conditions in which young people are responding.

Measurement of wellbeing is shaped by three overlapping traditions: hedonic or subjective wellbeing, typically indexed through affect and life satisfaction; eudaimonic or psychological wellbeing, which emphasises positive functioning, growth, purpose and self-acceptance; and social wellbeing, which foregrounds social integration, contribution, coherence, actualisation, and acceptance [20]. These traditions are related, but not interchangeable, and recent reviews confirm that overall wellbeing remains conceptually heterogeneous and is still measured using partly divergent frameworks, most of which were developed and validated in high-income settings [20–23]. High-income settings are more often characterised by individualistic cultures, which may place greater emphasis on eudaimonic than social wellbeing [17,24].

Promoting positive mental health remains essential for improving ALHIV’s wellbeing and long-term outcomes [17,25]. Intervention benefits may manifest as gains in belonging, recognition, future orientation, self-worth, or treatment confidence, even when distress remains [17,24]. We undertook a range of initiatives in collaboration with ALHIV to co-design a youth-focused, locally relevant quantitative positive psychology instrument; the Zvandiri Character Strength (ZCS) scale. The ZCS is a combination of learned and learnable attributes, virtues, skills, habits or capabilities that enable ALHIV to lead better lives.

Here, we report on our challenges and learnings to inform future initiatives. We frame the ZCS scale development process as a methodological case study within the psychometrics, patient-reported outcome measures, and wellbeing measurement literatures. The contribution is not to present relational wellbeing, response-process issues, social comparison, response shift, construct clarity or cross-cultural equivalence as new concerns; these are already well-established [26–30]. Rather, the ZCS case shows how these concerns became practically consequential when a relational HIV intervention in Zimbabwe required an interpretable positive wellbeing outcome measure.

## Methods

### Study overview

Fig 1 outlines the steps in the ZCS scale development process. For activities involving human subjects, we recruited participants from 1 March 2023 to 30 June 2025. For clarity, we use “adolescents” to refer to 13–19 year-olds and “young people” to 20–24 year-olds who took part in the various activities. Age-related considerations informed item wording, cognitive probing, and interpretation, with an emphasis on simple language, local meaning, and age-appropriate comprehension.

**Fig 1:**
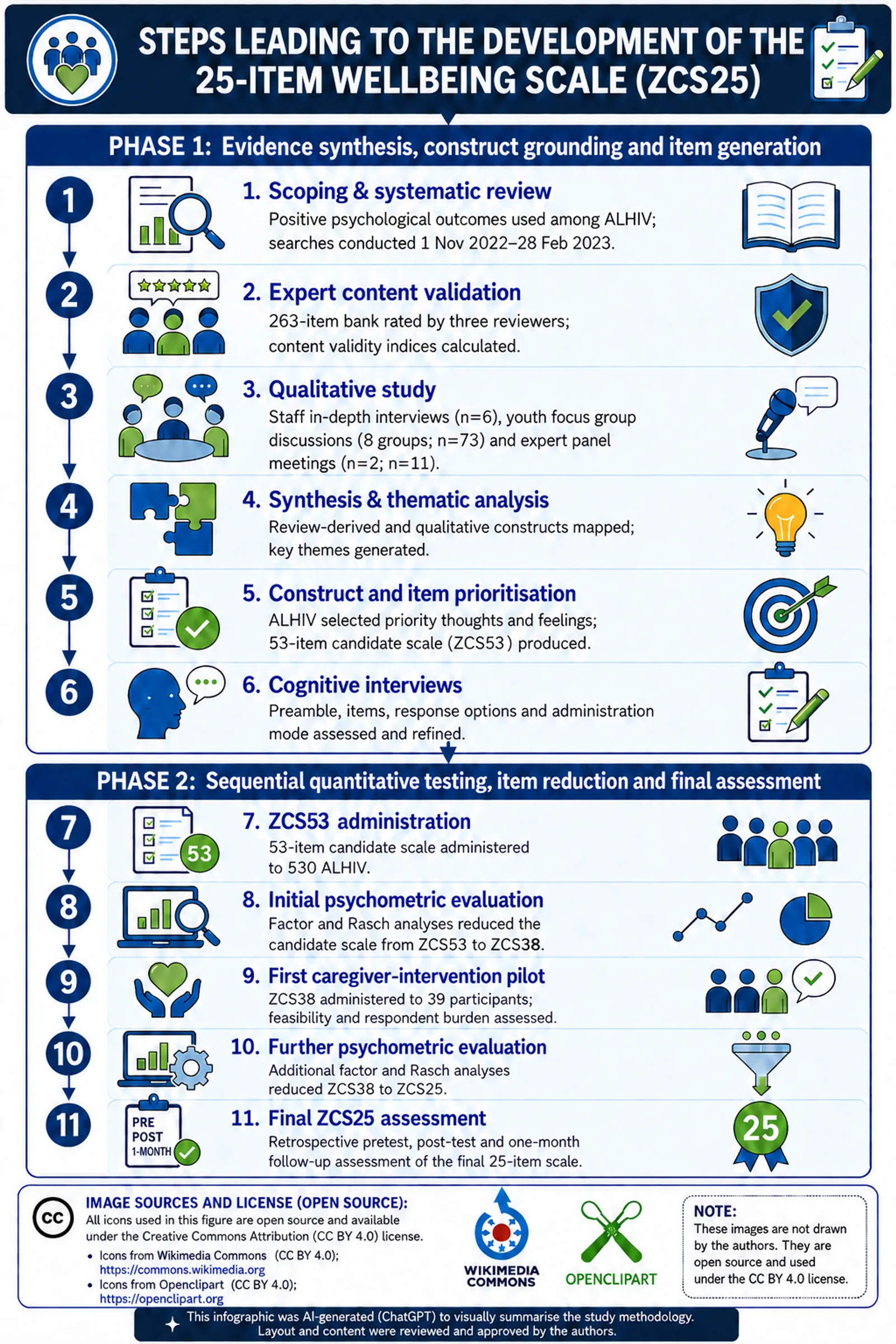
Steps leading to the 25-item wellbeing scale (ZCS 25).

We began by conducting a scoping and systematic review of positive psychological outcomes used in ALHIV (Fig 1**, step 1**) [17,31]. In brief, we searched relevant databases for articles published between 1 November 2022 and 28 February 2023. We initially planned to only extract items from outcome measures with robust validity and reliability. However, only a few outcome measures had been properly adapted and validated for use in low- and middle-income countries [17]. Consequently, we adopted a pragmatic approach by selecting items from measures with apparent content validity and using thematic analysis to identify and group conceptually equivalent or duplicate items, resulting in an initial bank of 263 items. Afterwards, three reviewers independently rated each item for relevance on a four-point scale, with ratings of 3 or 4 considered relevant. We assessed content validity using the item-level content validity index (I-CVI) and scale-level indices (S-CVI/Ave and S-CVI/UA), using modified kappa to adjust for chance agreement. Items with I-CVI = 1.00 were retained, those with I-CVI = 0.67 were revised, and those with I-CVI ≤ 0.33 were considered for removal; repeated or incomplete entries were excluded (Fig 1**, step 2**) [32].

We then conducted a complementary qualitative study that included six in-depth interviews with Zvandiri staff, eight focus group discussions with Zvandiri adolescents and young people (n = 73), and two expert panel meetings with Zvandiri adolescents and peer supporters (n = 11) (Fig 1**, step 3**). We applied narrative synthesis to map the key constructs emerging from the scoping review and conducted interpretive thematic analysis to generate themes from the qualitative data (Fig 1**, step 4**). We then worked with two separate groups of adolescents (n = 24) to identify the constructs/items they felt conveyed the most important thoughts or feelings that Zvandiri should aim for to help them think and feel their best (Fig 1**, step 5**). We consolidated both groups’ rankings, captured discussions of their thought processes, and analysed them qualitatively. The final product was a 53-item scale spanning 14 constructs (S1 Text). Items were scored on a Likert scale with responses: ‘strongly disagree’, ‘disagree’, ‘agree’, and ‘strongly agree’.

Next, we conducted cognitive interviews (n = 20) with ALHIV aged 13–19 years. Discussions were held in Shona or English, and used verbal probing [33] to assess comprehension of the scale’s preamble, whether respondents understood the items as intended, whether the response options worked as intended, and the preferred mode of scale administration (Fig 1**, step 6**). Findings informed revisions to the preamble, item wording, response format, and scale administration mode.

We then administered the 53-item scale to 530 ALHIV using an interviewer-administered card-ranking process (Fig 1**, step 7**). Participants included 240 Zvandiri beneficiaries and 290 ALHIV accessing treatment at a Harare tertiary facility who had not previously been in contact with Zvandiri. We included the latter to broaden the psychometric testing sample and increase response variability, but not necessarily as a formal comparison group. We subsequently held a group discussion with purposively-selected adolescents (n = 12) to explore emerging issues.

We used both factor and Rasch analyses to reduce the scale to 38 items (Fig 1**, step 8**). Next, we administered the ZCS 38 scale to 39 of 40 adolescents who had participated in a pilot intervention exploring whether targeting caregivers improved a young person’s relational environment and, therefore, their wellbeing, to assess the scale’s feasibility, particularly regarding respondent burden (Fig 1**, step 9**, one participant was unavailable). Following additional factor and Rasch analyses, we further reduced the items to 25, hence the ZCS 25 scale (Fig 1**, step 10**).

Lastly, we administered the ZCS 25 scale to eight adolescents who had participated in a second caregiver-intervention pilot. Participants first completed a retrospective pretest, in which they were asked to think back to before they began the programme and rate their wellbeing at that time; after a 30-minute break, they completed the same items based on their current wellbeing (post-test) **(Fig 1, step 11).**

### Psychometric analyses

Analyses were iterative and examined item performance, dimensionality, response-category functioning, and reliability. Exploratory factor analysis of the 53- and 38-item versions used maximum likelihood with oblique rotations. Rasch analysis assessed item fit, residuals, and threshold ordering. Items were removed based on weak item-total correlations, poor Rasch fit, disordered thresholds, low loadings, cross-loadings, redundancy, and conceptual coverage. We assessed internal consistency using Cronbach’s alpha and intraclass correlation coefficients, interpreted alongside content validity and response-process evidence.

### Ethics statement

This study was approved by the Medical Research Council of Zimbabwe (#A/2860). We obtained written informed consent from participants aged ≥18 years and assent, plus parental consent, from those <18 years. We removed names and other personal identifiers from transcripts and other data sources before analyses. Participants received US$5 as reimbursement for bus fare.

## Results

### Participant characteristics

Participant characteristics varied across study phases (Table 1). Zvandiri staff interviewees were aged 30–40 years, and comprised programme managers and counsellors. Youth focus group discussion (FGD) participants were aged 15–24 years, with an approximately equal sex distribution. Cognitive interview participants were aged 13–19 years. The largest sample was the ZCS-53 psychometric evaluation group (n = 530, mean age 16.3 years (SD 2.1); 342 (64.5%) female) (Table 1).

**Table 1:** Participant characteristics across phases of ZCS 25 development.

Study phase	Variable	Attribute/category	Frequency, n (%)/ range
**Qualitative study – staff interviews (n = 6)**	Age, years	Range	30-40
	Sex	Male	3
		Female	3
	Professional role	Programme managers	3
		Counsellors	3
**Qualitative study – youth FGDs (n = 73)**	Age, years	Range	15-24
	Sex	Male	36 (50)
		Female	37 (50)
**Expert panel meetings (n = 11)**	Professional/expert background	Psychometrics & global mental health epidemiology	1
		Social Sciences	1
		Clinical Psychology	1
		Lived experiences experts	8
**Cognitive interviews (n = 20)**	Age, years	Range	13-19
	Sex	Male	10 (50)
		Female	10 (50)
**Psychometric evaluation – ZCS 53 (n = 530)**	Age, years	Mean (SD)	16.3 (2.1)
	Sex	Male	188 (35.5)
		Female	342 (64.5)
**Caregiver-intervention pilot – ZCS 38 (n = 39)**	Age, years	Range	13-19
	Sex	Male	15 (38%)
		Female	24 (62%)
**Final ZCS 25 assessment (n = 8)**	Age, years	Range	13-17
	Sex	Male	5
		Female	3

***Notes.*** *Values are presented as n (%) unless otherwise indicated. For cells with counts <10, absolute values are reported without percentages to avoid misleading interpretation. Continuous variables are presented as mean (SD) or range, where appropriate. Percentages may not total 100% because of rounding and/or missing data.*

### Review findings

The scoping and systematic review has been published elsewhere [17]. We identified 15 positive psychological constructs used to assess ALHIV: body appreciation, confidence, coping, flourishing, meaningfulness, personal control, positive outlook, resilience, self-management, self-compassion, self-concept, self-efficacy, self-esteem, self-worth, and transcendence [17]. Resilience, self-esteem, and self-efficacy were the most measured constructs [17]. Although the scales had been used in Africa, all but one were developed in high-income settings, and were not properly translated and validated before use in Africa.

### Content validity findings

Of 263 candidate items, we included 217 with complete ratings from all three reviewers; we excluded 45 duplicate items and one item with a non-numeric rating. The overall S-CVI/Ave was 0.730, and S-CVI/UA was 0.369. Eighty items (36.9%) achieved unanimous agreement (I-CVI = 1.00), 100 (46.1%) had an I-CVI of 0.67 and were flagged for revision, and 37 (17.1%) had an I-CVI ≤ 0.33 and were considered for removal. Construct-level S-CVI/Ave values were highest for gratitude and perseverance (0.889 each) and lowest for flourishing (0.587) (S1 Data).

### Qualitative findings

The initial qualitative piece, which complemented the scoping and systematic review, identified six dominant constructs emerging from the data: connectedness, hope, self-acceptance, self-efficacy, purpose, and flourishing. That initial piece and subsequent ones provided insights on the constructs’ context and nuances. For example, it emerged that, in this setting, “self-acceptance” emerges in relation to others and focuses on accepting oneself through participation in a community (i.e., I accept myself within this community). Further, adolescents mentioned that they considered their own wellbeing in comparison to others in the community (i.e., I am doing well compared to others). As one participant remarked, *‘Yes, I am positive [HIV], but I have a bright future. I can even compare myself with others... I can say that I am not the young person with the worst challenges…”* (Participant #2 ZCS workshop 2). This has important implications for measurement as wellbeing is not only relational but also evaluated in comparison to other possibilities, i.e., (It could be worse, as I see it is for others; therefore, for me it is not too bad - even though I am struggling with my wellbeing).

In all discussions, adolescents and young people highlighted the fluctuating nature of all constructs and cautioned against assuming their continuous presence. They explained this in part because precarious contextual conditions continuously threaten their wellbeing. Finally, discussions highlighted how language around mental health and wellbeing is constrained. For example, it was difficult to differentiate between some constructs (e.g., hope and optimism) or at the very least, to find near-equivalent indigenous terms. These factors have implications for measuring the Zvandiri Character Strength and wellbeing more generally.

### Scale administration findings

Data from the first 530 participants who completed the 53‑item scale showed consistently high endorsement across all items. Item‑level proportions for strongly agree ranged from 34% to 67%, while combined agree/strongly agree responses ranged from 81% to 98%. These figures reflect item-level endorsement patterns rather than participant‑level estimates, and clear ceiling effects across the scale. (Reporting both strongly agree and the combined category highlights the distinction between extreme endorsement and overall positive endorsement).

A scree plot identified four factors, but inter-item correlations were low and multiple items cross-loaded across factors. Rasch analysis of polytomous responses showed that the response thresholds were disordered (See S2 Data for detailed analyses, including item-level response distributions, factor loadings, Rasch diagnostics, threshold maps, and item-retention decisions). Since the ordinal responses yielded disordered thresholds, we used binary responses (agree/disagree) for the ZCS-38 scale after consulting with adolescents. Thirty-nine adolescents completed the ZCS-38 (reduced from 53 items). We identified order-related ceiling effects. For the first 15 scale items, agreement ranged from 80% to 90%; for items 16–38, agreement ranged from 92% to 100%. This indicated questionnaire fatigue and unreliable responses.

As stated above, we held a group discussion with adolescents to investigate the causes of the ceiling effects. The adolescents told us the statements were all positive affirmations that encouraged agreement, and that, in the presence of an interviewer, they were concerned about being judged if they did not report positive responses. We added a preamble explaining that there was no need to give responses which pleased the interviewer. This helped us interpret high scores as potentially reflecting a mixture of genuine positive appraisal, encouragement from the item wording, social desirability, demand characteristics, and concern about how disagreement would be judged (36, 37). To improve response variability, we reverted to a 4-item Likert scale of responses, using ‘never’, ‘seldom’, ‘some of the time’, and ‘most of the time’ rather than strength of agreement. We benchmarked the responses to ‘in the past two weeks’. To minimise questionnaire fatigue, we reduced the number of items to 25 (ZCS 25).

As stated earlier, eight adolescents completed a retrospective pretest (Phase 1), and a post-test (Phase 2) of the ZCS 25 scale with the new response options, and five of them completed the ZCS 25 scale again a month later (Phase 3). Scores were calculated as never = 0, seldom = 1, some of the time = 2, most of the time = 3 (range 0–75). Retrospective pretest and post-test responses fluctuated, making the results difficult to interpret (Fig 2). We expected the Phase 1 score to be lower than Phase 2, and Phase 2 to be similar to Phase 3, but the data did not support this. Two participants reported that their wellbeing after the intervention was considerably lower than it had been retrospectively, but in qualitative discussion, they said that, on the contrary, the intervention had positively impacted their wellbeing. We interpreted this discordance as a limitation of the scale. Ceiling effects continued; four participants reported scores of 70–75.

**Fig 2:**
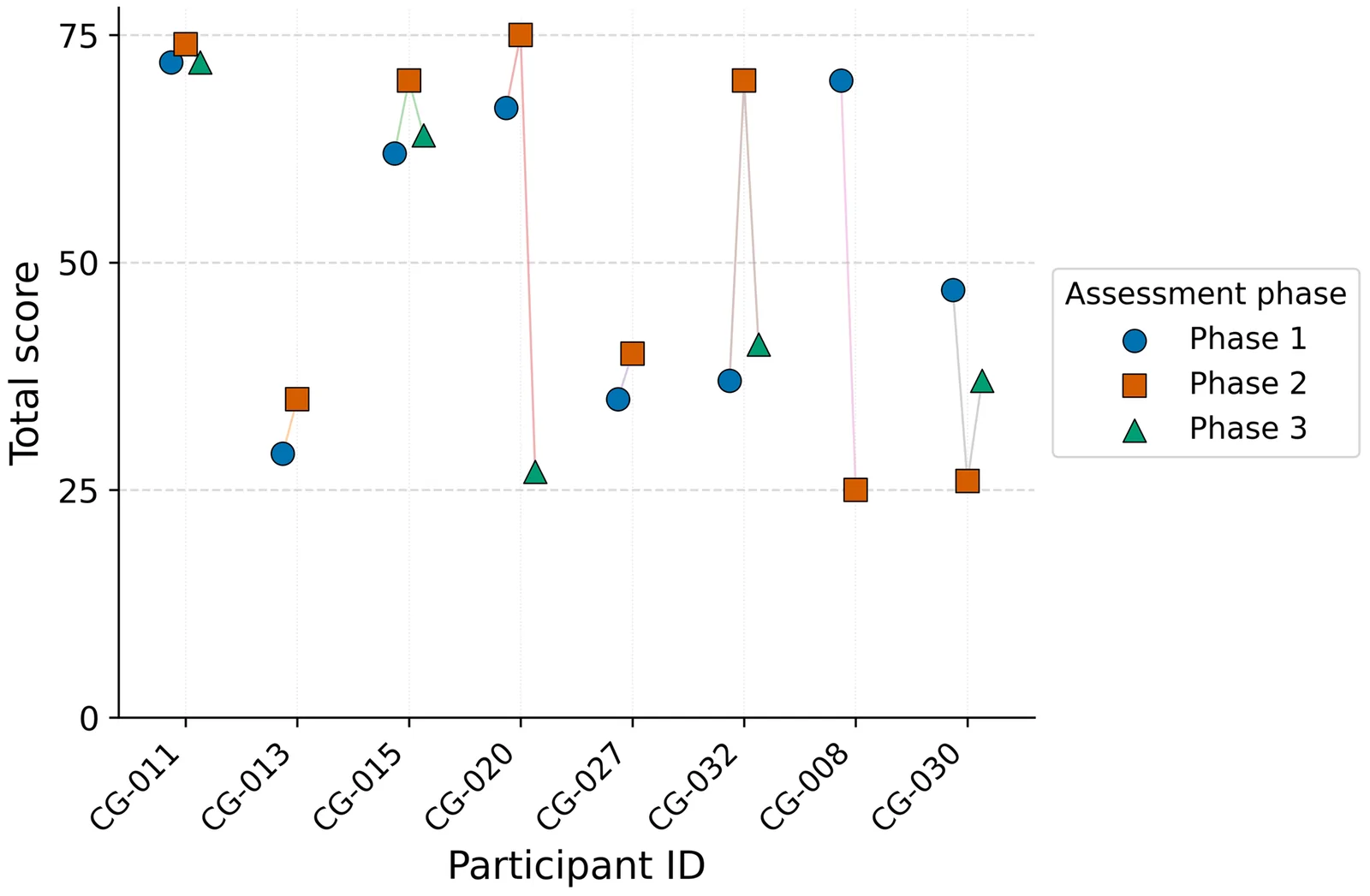
ZCS scale scores for three phases.

Taken together, persistent ceiling effects, fluctuating retrospective ratings, and discordance between qualitative accounts and ZCS 25 scores did not support using the ZCS 25 as an outcome measure without substantial further development. We, therefore, did not proceed to larger-scale quantitative validation at this stage.

## Discussion

We aimed to develop a contextually relevant positive psychological outcome measure for adolescents living with HIV (ALHIV) in Zimbabwe, with potential application to Eastern and Southern Africa more broadly. We followed recognised best-practice standards for patient-reported outcome measure development and validation, including a systematic review and some qualitative interviews to generate candidate items [26]. However, the measure did not achieve the reliability and validity required for confident use. Here, we share lessons learned and recommendations for future studies.

One possible explanation lies in the alignment between the construct definition, item content, response process, and intended score interpretation. We initially explored whether the ZCS could support a single score for positive wellbeing or “character strength”, but the development process brought together several conceptually adjacent yet non-interchangeable domains. In the qualitative work with adolescents, six dominant constructs emerged: connectedness, hope, self-acceptance, self-efficacy, purpose, and flourishing. These domains span social experience, affective state, future orientation, motivational capacity and coping, and therefore, may not function as indicators of a single homogeneous latent trait.

The lack of a coherent factor and Rasch structure should not be interpreted as evidence that wellbeing among ALHIV cannot be measured, nor that reflective measurement is inherently inappropriate. Rather, it suggests that the proposed total score was not supported by the available evidence. This may reflect restricted variance due to ceiling effects, disordered response categories, interviewer/social desirability effects and insufficiently specified score interpretation [27,34–37]. Future work should therefore return to construct pruning, response-process validation and domain-level testing before attempting to justify a single ZCS total score.

The second issue is the construct jangle problem. The broader wellbeing and positive psychology literature includes many overlapping labels: self-worth, self-esteem, self-concept, confidence, self-efficacy, resilience, coping, hope, optimism, perseverance, and flourishing. These are not identical constructs, but they are often treated as if their boundaries are self-evident. The jingle-jangle problem is well established in psychological measurement: different labels may be used for highly similar constructs, while similar labels may conceal different constructs [37,38]. More recent critiques of psychological measurement also warn that weak construct specification and proliferation of partially overlapping measures can undermine validity before statistical modelling even begins [27]. Constructs identified through the systematic review should not automatically be treated as a coherent conceptual framework; rather, they may reflect underlying conceptual heterogeneity. Careful definition, differentiation, and pruning grounded in the lived experiences of ALHIV are therefore required before item generation and structural testing to produce measures that are both conceptually coherent and contextually fit.

Third, the factor and Rasch analyses results may have been compromised by response distributions. If most respondents cluster at the positive end of most items, the correlation matrix may not contain sufficient variation to recover structure, even if meaningful domains exist. Fourth, is the issue of response-process validity. Adolescents’ comments that the items sounded like positive affirmations, and that they worried about being judged by an interviewer, are response-process evidence. In this case, observed responses may have been shaped by social desirability, demand characteristics, acquiescence, interviewer effects, politeness norms, or loyalty to a valued programme, as well as by underlying wellbeing. Classic work on social desirability and demand characteristics is directly relevant here [39,40], as is the wider survey-methods literature on how respondents interpret questions and construct answers in context [33,41,42].

Fifth, cross-cultural survey research shows that response styles, including acquiescence and extreme responding, can vary across cultural and survey contexts [35,43,44]. There is also directly relevant evidence from adolescent and HIV-related research in Eastern and Southern Africa that the mode of administration and privacy can shape self-reports [45–47]. Although these studies concern sexual behaviour rather than wellbeing, they are relevant because they demonstrate that adolescent self-report in HIV-related and sexual-health research is vulnerable to administration mode, privacy, perceived judgement, and social desirability.

Another key issue is the centrality of relationality. In Africa, the philosophy of *Ubuntu* (humanity) positions personhood as fundamentally constituted through relationships with others [48,49]. *Ubuntu* therefore frames human flourishing not as an individual possession, but as something realised through mutual recognition and harmonious coexistence. From this perspective, wellbeing is not reducible to an internal psychological state located solely within the individual. Instead, it is both relational and socially embedded, and, as stated earlier, this has implications for measurement.

Our findings highlight a tension between capturing wellbeing as lived and measuring intervention-relevant change. Qualitative and participatory activities suggested that adolescents experience wellbeing as uneven, contextually-situated and often simultaneous with distress; earlier Zvandiri work similarly found that young people represented feelings as crooked rather than linear trajectories [50,51], reflecting lives in which adversity continued but support could provide comfort, connection, respite, and manageability. Further, adolescence is marked by rapid physical, hormonal, neural, psychosocial, cognitive, and emotional transitions [52]. Future ZCS work should therefore clarify whether the intended measure is capturing average wellbeing over a defined period, variability across contexts, or change in specific intervention mechanisms, and should consider context-specific items, repeated assessment, visual trajectory methods or mixed-method interpretation where fluctuation and appraisal are central to the construct.

To interpret our experiences of ZCS scale development, we draw on conceptualisations of wellbeing as multidimensional, relational, and dynamic [22,53–55]. The contribution of the ZCS process is more specific: it shows how these established issues became practically consequential when trying to develop an outcome measure for a relational intervention with ALHIV in Zimbabwe. Future work should therefore move beyond simple adaptation of imported, individual-level measures, but not because quantitative measurement is inappropriate. Rather, the measurement approach needs to be aligned with the intervention theory of change and the intended score interpretation. If the aim is to capture wellbeing as lived, then methods may need to attend to fluctuation, context, relationships and narrative meaning, using qualitative, vignette-based, visual, diary or ecological momentary assessment (EMA)-informed approaches where appropriate [56–58]. If the aim is to evaluate intervention benefit, however, the measure must also be capable of detecting theorised change in specific domains that an intervention seeks to influence, such as connectedness, self-worth, hope, treatment confidence, relational safety, or manageability. These aims are related but not identical. The practical lesson is that future ZCS measurement must decide what kind of change it is trying to detect, at what level, over what time frame, and using which combination of quantitative and qualitative methods.

Importantly, our results should not be read as evidence that positive wellbeing cannot be measured among ALHIV in Zimbabwe, nor that rigorous psychometric methods are irrelevant. We already have good examples to show that local concepts of self, family, spirituality, belonging and wellbeing can be operationalised psychometrically, but only when construct development precedes scoring. These include for example, the Shona Symptom Questionnaire in Zimbabwe, which developed an indigenous measure of common mental disorders from local idioms of distress [59]; the Hua Oranga, a Maori mental health outcome measure grounded in Maori models of health [60]; and the mixed-methods youth mental health scale development in Rwanda [61]. In that sense, the main contribution of this study is methodological as much as it is substantive.

### Strengths and limitations

A strength of our approach is that we employed recognised best-practice standards for patient-reported outcome measure development and validation, including a systematic review and allied qualitative and participatory components to generate candidate items. A potential limitation is the small number of adolescents who took part in the three phases of ZCS 25 completion, necessitated by the desire to conduct an in-depth exploration of ceiling effects. We also did not compare different modes of scale administration. Future initiatives could include fuller item- and domain-level documentation, participant-characteristic reporting, administration-mode testing, domain-specific analyses, and systematic response-process evidence.

The retrospective pretest may also have been affected by recall bias, current mood and response shift. In addition, interviewer administration may have introduced social desirability, demand characteristics, politeness norms, and concern about judgement. Future studies should compare interviewer-administered and anonymous self-completion modes, include systematic response-process probes, report phase-specific participant characteristics and attrition, and test domain-specific scoring before attempting a single total score.

## Conclusion

We implemented a series of initiatives to collaboratively develop a youth-focused, locally relevant, quantitative instrument to evaluate the Zvandiri Character Strength. Our experiences, consistent with those from similar settings [19,62], underscore the necessity of appropriately tailoring (wellbeing) scales originally designed elsewhere prior to their application to this context. Future ZCS measurement initiatives should, therefore, move beyond the simple adaptation of imported wellbeing scales and begin with a clear definition of each construct, locally grounded language, response-process evidence, and an explicit decision about what the measure is intended to capture: lived wellbeing, domain-specific positive outcomes, or intervention-relevant change. This decision should guide item content, recall period, response options, administration mode, scoring, and the balance between quantitative and qualitative evidence.

## Supporting information

S1 Text 53-item scale spanning 14 constructs. (DOCX)

S1 Data Panel of experts’ content validation results. (XLSX)

S2 Data Detailed psychometric evaluation results. (DOCX)
