# Supplementary material for: Rethinking wellbeing measurement: Learning from a scale development study with adolescents living with HIV in Zimbabwe
Source: PLOS Glob Public Health. 2026 Sep 30;6(9):e0006845. doi: 10.1371/journal.pgph.0006845 (PMC13626463; doi:10.1371/journal.pgph.0006845)
Supplement: Supplementary file 1 [file pgph.0006845.s001.docx]

**S1 File: 53-item scale spanning 14 constructs**

**Self-Acceptance**

1.       I feel I am a valuable person even when other people don’t like me.

2.       I love myself just the way I am.

3.       Living with HIV does not define who I am.

**Self-esteem**

4.       I am proud of myself.

5.       I can do things as well as other people.

6.       I feel that I'm just as worthy as other people.

**Self-efficacy**

7.       Living with HIV has not affected my ability to do what I want in life.

8.       I have what it takes to achieve my goals.

9.       I am confident in myself.

**Adherence Self-efficacy**

10.       I am confident that I can manage my treatment.

11.       I try to make a way to attend my clinic appointment, even when I have some challenges doing so.

12.       I do things to try and stay healthy (e.g. eat healthy foods, drink water, exercise).

**Hope**

13.       When I grow up I will have a good family life.

14.       When I grow up, I will have the life I want.

15.       I will have a long life.

16.       I will be able to take care of myself.

17.   I feel good about my future.

18.   Living with HIV will not stop me from achieving my goals.

**Optimism**

19.       I am optimistic about my future.

20.       I believe that things will work out, no matter how difficult they seem.

21.       If I feel down, I always think about what is good in my life.

**Purpose in Life**

22.       Getting an education is important to me.

23.       I have a reason for living.

24.       I work hard to achieve my goals.

**Connectedness**

25.       I feel comfortable around my family.

26.       I have friends that I trust.

27.       I feel like I belong in my community.

28.       My family stands by me during difficult times.

29.       I feel supported by the clinic/hospital staff.

**Flourishing**

30.       I lead a purposeful and meaningful life.

31.       I am engaged and interested in my daily activities.

32.       I am competent and capable in the activities that are important to me.

33.       I have been feeling useful.

34.       I have been dealing with problems well.

**Resilience**

35.       I work well with people my age.

36.       I feel good when I am at school.

37.       I feel like I have enough support.

38.       I see life challenges as a chance for me to learn and grow.

39.       I am confident that I can overcome the challenges in life.

**Coping**

40.       When I have a problem, I think about different ways to solve the problem.

41.       I avoid my problems.

42.       When I think of living with HIV, I remind myself that there are others who are like me.

**Perseverance**

43.       I move out of my comfort zone to achieve my goal.

44.       I am able to handle whatever comes my way.

45.       I finish whatever I begin.

**Transcendence**

46.       I enjoy the process of striving toward excellence in what matters.

47.       What matters most to me in life is the contribution I make to society.

48.       I can separate myself from my thoughts and feelings.

49.       I have the sense that I am fully aware of what is going on around me and inside me.

50.   I focus on discovering the potential meaning in every situation.

**Mastery**

51.       I can do anything when I put my mind to it.

52.       What happens to me in the future mostly depends on me.

53.       My life is in my hands and I am in control of it.
