# Supplementary material for: Rethinking wellbeing measurement: Learning from a scale development study with adolescents living with HIV in Zimbabwe
Source: PLOS Glob Public Health. 2026 Sep 30;6(9):e0006845. doi: 10.1371/journal.pgph.0006845 (PMC13626463; doi:10.1371/journal.pgph.0006845)
Supplement: Supplementary file 2 [file pgph.0006845.s003.docx]

# S3 File: Detailed psychometric analysis

## Round 1 Psychometric evaluation analysis

### Stage 1 – testing for assumptions

##### Normality and identity matrices

Data were not normally distributed (W=0.967, df=264, p< .0001), a violation of EFA assumptions. However, the data were factorable, as they exhibited adequate sampling adequacy (Kaiser-Meyer-Olkin Measure of Sampling Adequacy = .914), and the Bartlett Test of Sphericity was statistically significant (χ2 (df=1378) =5700.0, p<0.001).

**Quick comment:** Although the data were not normally distributed, they met the requirements for factor analysis.

##### Correlations

Most items correlated well with the total score (See Table 1). Item A03 had the lowest correlation (r=0.22), with item K41 recording the second lowest correlation (r=0.36). The inter-item correlations are illustrated in Table 7. Generally, there was no evidence of multicollinearity. However, items did not correlate reasonably, with a few correlations greater than 0.3 (See Appendix 1).

**Quick comment**: The low inter-item correlations raise concerns. Since the ZCS is intended to measure positive psychological constructs, items should correlate strongly; however, few items reached the r ≥ 0.3 threshold.

Table 1: Item-total correlations

| **Item** | **ITC** | **Item** | **ITC** |
| --- | --- | --- | --- |
| A01. I feel I am a valuable person even when other people don't like me. | 0.50 | H26. I have friends that I trust. | 0.45 |
| A02. I love myself just the way I am. | 0.41 | H27. I feel like I belong in my community. | 0.61 |
| A03. Living with HIV does not define who I am. | 0.22 | H28. My family stands by me during difficult times. | 0.56 |
| B04. I am proud of myself. | 0.42 | H29. I feel supported by the clinic/hospital staff. | 0.45 |
| B05. I can do things as well as other people. | 0.44 | I30. I lead a purposeful and meaningful life. | 0.64 |
| B06. I feel that I'm just as worthy as other people. | 0.41 | I31. I am engaged and interested in my daily activities. | 0.57 |
| C07. Living with HIV has not affected my ability to do what I want in life. | 0.41 | I32. I am competent and capable in the activities that are important to me. | 0.61 |
| C08. I have what it takes to achieve my goals. | 0.49 | I33. I have been feeling useful. | 0.52 |
| C09. I am confident in myself. | 0.54 | I34. I have been dealing with problems well. | 0.55 |
| D010. I am confident that I can manage my treatment. | 0.56 | J35. I work well with people my age. | 0.63 |
| D011. I try to make a way to attend my clinic appointment, even when I have some | 0.50 | J36. I feel good when I am at school. | 0.50 |
| D012. I do things to try and stay healthy (e.g. eat healthy foods, drink water, | 0.51 | J37. I feel like I have enough support. | 0.56 |
| E13. When I grow up I will have a good family life. | 0.53 | J38. I see life challenges as a chance for me to learn and grow. | 0.57 |
| E14. When I grow up, I will have the life I want. | 0.53 | J39. I am confident that I can overcome the challenges in life. | 0.60 |
| E15. I will have a long life. | 0.49 | K40. When I have a problem, I think about different ways to solve the problem. | 0.59 |
| E16. I will be able to take care of myself. | 0.57 | K41. I avoid my problems. | 0.36 |
| E17. I feel good about my future. | 0.59 | K42. When I think of living with HIV, I remind myself that there are others who | 0.53 |
| E18. Living with HIV will not stop me from achieving my goals. | 0.47 | L43. I move out of my comfort zone to achieve my goal. | 0.58 |
| F19. I am optimistic about my future. | 0.58 | L44. I am able to handle whatever comes my way. | 0.57 |
| F20. I believe that things will work out, no matter how difficult they seem. | 0.57 | L45. I finish whatever I begin. | 0.58 |
| F21. If I feel down, I always think about what is good in my life. | 0.53 | M46. I enjoy the process of striving toward excellence in what matters. | 0.58 |
| G22. Getting an education is important to me. | 0.49 | M47. What matters most to me in life is the contribution I make to society. | 0.53 |
| G23. I have a reason for living. | 0.56 | M48. I can separate myself from my thoughts and feelings. | 0.53 |
| G24. I work hard to achieve my goals. | 0.59 | M49. I have the sense that I am fully aware of what is going on around me and in | 0.42 |
| H25. I feel comfortable around my family. | 0.53 |  |  |

#### Rasch-based item reduction

Item fit is the extent to which a specific item satisfies the requirements of the Rasch model, i.e. the extent to which it is reflective of the domain under investigation. In the present study, items with a perfect fit indeed measure positive psychological constructs. Items K41 and A03 were candidates for removal as given residuals ≥ 2, as evidenced by infit and outfit statistics (Table 2).

**Quick comment:** Based on Rasch's analysis, items in the bottom half of Table 2 are candidates for inclusion as they exhibited reasonable fit. Items K41 and A03 were dropped for the rest of the analysis based on correlations and fit indices.

Table 2: Rasch item fit statistics

#### Stage 2 – Factor extraction

Four (4) unique factors were extracted per Kaiser criteria, i.e. eigenvalues≥1 (Table 3). Unfortunately, the screen plot results were inconclusive but suggest a four-factor solution.

Table 3: ZCS factor extraction

| Total Variance Explained | |
| --- | --- |
| Factor | Rotation Sums of Squared Loadings^a^ |
| 1 | 11.072 |
| 2 | 9.380 |
| 3 | 10.730 |
| 4 | 7.559 |

Figure 1: Scree plot for ZCS

**Quick comment:** There can be as many factors as items. Given the divergent Kaiser extraction and scree plot outputs, there is a need to iteratively remove items and inspect the resultant factor matrices.

#### Stage 3 – Factor rotation and interpretation

Four factors were retained upon inspection of both structure and pattern matrices (See Table 4 ). Factors underwent Promax (oblique) rotation method to improve interpretability. Furthermore, item loadings were high, ranging from 0.312 to 0.779. The structure matrix shows multiple cross-loadings for almost all items across the four factors.

Table 4: ZCS matrices

| **Pattern Matrix** | | | | | **Structure Matrix** | | | | |
| --- | --- | --- | --- | --- | --- | --- | --- | --- | --- |
|  | **Factor** | | | | **Factor** | | | | |
| Item | 1 | 2 | 3 | 4 | Item | 1 | 2 | 3 | 4 |
| l44 | .779 |  |  |  | l44 | .722 | .406 | .462 | .318 |
| m47 | .639 |  |  |  | l43 | .680 | .405 | .530 |  |
| l43 | .632 |  |  |  | l45 | .654 | .347 | .490 | .416 |
| i34 | .605 |  |  |  | m47 | .643 | .406 | .424 | .380 |
| m48 | .605 |  |  |  | i34 | .633 | .369 | .466 |  |
| l45 | .597 |  |  |  | m48 | .621 | .372 | .476 |  |
| m50 | .565 |  |  |  | n52 | .618 | .423 | .473 |  |
| n52 | .552 |  |  |  | n51 | .615 | .546 | .600 |  |
| m49 | .527 |  |  |  | j39 | .614 | .385 | .542 | .302 |
| j39 | .467 |  |  |  | h27 | .601 | .486 | .488 | .361 |
| f21 | .455 |  |  | .344 | k40 | .596 | .473 | .520 |  |
| h27 | .451 |  |  |  | m49 | .559 |  | .421 |  |
| k40 | .450 |  |  |  | f21 | .558 |  | .434 | .496 |
| h26 | .364 |  |  |  | m50 | .544 | .328 | .341 | .329 |
| n51 | .356 |  |  |  | i33 | .502 | .486 | .431 | .340 |
| i33 | .312 |  |  |  | h26 | .439 |  | .350 | .333 |
| b04 |  | .742 |  |  | b04 | .326 | .629 | .346 |  |
| a02 |  | .664 |  |  | a02 | .335 | .624 | .380 | .309 |
| b06 |  | .586 |  |  | b06 | .321 | .620 | .443 | .353 |
| e18 |  | .525 |  |  | e18 | .401 | .598 | .431 | .362 |
| c07 |  | .471 |  |  | d10 | .478 | .590 | .502 | .419 |
| a01 |  | .420 |  |  | k42 | .505 | .582 | .578 |  |
| b05 |  | .413 |  |  | b05 | .336 | .554 | .499 |  |
| d10 |  | .396 |  |  | a01 | .311 | .522 | .459 |  |
| k42 |  | .366 |  |  | c07 | .312 | .514 | .374 |  |
| h28 |  |  | .670 |  | m46 | .598 | .507 | .670 |  |
| h29 |  |  | .541 |  | i32 | .561 | .521 | .658 | .308 |
| g23 |  |  | .514 |  | h28 | .475 | .358 | .639 | .369 |
| j38 |  |  | .509 |  | f19 | .511 | .509 | .631 | .463 |
| m46 |  |  | .473 |  | g23 | .433 | .534 | .625 | .337 |
| i32 |  |  | .466 |  | e16 | .458 | .583 | .618 | .610 |
| f19 |  |  | .443 |  | n53 | .527 | .462 | .601 | .306 |
| n53 |  |  | .420 |  | j38 | .513 | .380 | .594 |  |
| d11 |  |  | .392 |  | h29 | .417 | .425 | .586 | .329 |
| e14 |  | .395 |  | .453 | d11 | .418 | .484 | .544 |  |
| e16 |  |  | .370 | .410 | e14 | .464 | .570 | .419 | .616 |
| e13 |  | .335 |  | .378 | e13 | .483 | .563 | .471 | .567 |
| d12 |  |  |  | .352 | d12 | .419 | .495 | .491 | .523 |
| Extraction Method: Maximum Likelihood. Rotation Method: Promax with Kaiser Normalization. | | | | | | | | | |

**Quick comment:** Items with multiple cross-loadings are candidates for removal. However, multiple cross-loadings in the ZCS structure matrix may also imply interconnectedness among the scales.

#### Stage 4: Reliability analysis

The ZCS yielded a high internal consistency (α = .946). Deleting any of the items did not improve α, indicating high item reliability – **See Table 5**.

Table 5: ZCS scale internal consistency

| **Item** | **Scale Mean if Item Deleted** | **Scale Variance if Item Deleted** | **Corrected Item-Total Correlation** | **Cronbach's Alpha if Item Deleted** |
| --- | --- | --- | --- | --- |
| a01 | 128.64 | 155.542 | .447 | .945 |
| a02 | 128.67 | 155.196 | .476 | .945 |
| b04 | 128.72 | 154.873 | .445 | .945 |
| b05 | 128.68 | 154.624 | .492 | .945 |
| b06 | 128.67 | 155.101 | .494 | .945 |
| c07 | 128.76 | 154.381 | .429 | .945 |
| d10 | 128.74 | 153.380 | .583 | .944 |
| d11 | 128.74 | 154.543 | .512 | .944 |
| d12 | 128.75 | 154.352 | .544 | .944 |
| e13 | 128.68 | 154.073 | .594 | .944 |
| e14 | 128.75 | 153.904 | .574 | .944 |
| e16 | 128.71 | 153.705 | .633 | .944 |
| e18 | 128.72 | 153.792 | .528 | .944 |
| f19 | 128.70 | 153.844 | .624 | .944 |
| f21 | 129.00 | 152.151 | .531 | .944 |
| g23 | 128.76 | 153.988 | .571 | .944 |
| h26 | 129.11 | 153.280 | .409 | .946 |
| h27 | 128.92 | 152.426 | .607 | .944 |
| h28 | 128.75 | 153.149 | .551 | .944 |
| h29 | 128.68 | 154.577 | .518 | .944 |
| i32 | 128.79 | 153.106 | .634 | .944 |
| i33 | 128.88 | 153.624 | .535 | .944 |
| i34 | 128.99 | 152.573 | .564 | .944 |
| j38 | 128.83 | 153.569 | .544 | .944 |
| j39 | 128.86 | 153.080 | .581 | .944 |
| k40 | 128.93 | 152.961 | .575 | .944 |
| k42 | 128.76 | 153.013 | .576 | .944 |
| l43 | 128.89 | 152.658 | .599 | .944 |
| l44 | 128.94 | 152.249 | .609 | .944 |
| l45 | 128.94 | 152.941 | .589 | .944 |
| m46 | 128.74 | 153.328 | .638 | .944 |
| m47 | 128.89 | 153.146 | .578 | .944 |
| m48 | 128.98 | 153.016 | .536 | .944 |
| m49 | 128.89 | 154.495 | .488 | .945 |
| m50 | 129.02 | 154.062 | .482 | .945 |
| n51 | 128.83 | 152.660 | .635 | .943 |
| n52 | 128.82 | 153.466 | .564 | .944 |
| n53 | 128.71 | 153.320 | .580 | .944 |

The ZCS four subscales exhibited high internal consistency, ranging from .778 to .900; this is evidence of high subscale reliability (Table 6). Subscale reliability is a function of the number of items, i.e., the greater the number of items, the more likely the higher the α.

Table 6: ZCS SUB-SACLES INTERNAL CONSISTENCY

| **Subscale** | **Items** | **# of items** | **α** | **ICC (95% CI)** |
| --- | --- | --- | --- | --- |
| **1** | l44 | **16** | **.900** | **.887; .912** |
|  | m47 |  |  |  |
|  | l43 |  |  |  |
|  | i34 |  |  |  |
|  | m48 |  |  |  |
|  | l45 |  |  |  |
|  | m50 |  |  |  |
|  | n52 |  |  |  |
|  | m49 |  |  |  |
|  | j39 |  |  |  |
|  | f21 |  |  |  |
|  | h27 |  |  |  |
|  | k40 |  |  |  |
|  | h26 |  |  |  |
|  | n51 |  |  |  |
|  | i33 |  |  |  |
| **2** | b04 | **9** | **.824** | **.801; .846** |
|  | a02 |  |  |  |
|  | b06 |  |  |  |
|  | e18 |  |  |  |
|  | c07 |  |  |  |
|  | a01 |  |  |  |
|  | b05 |  |  |  |
|  | d10 |  |  |  |
|  | k42 |  |  |  |
| **3** | h28 | **9** | **.852** | **.832; .870** |
|  | h29 |  |  | .541 |
|  | g23 |  |  | .514 |
|  | j38 |  |  | .509 |
|  | m46 |  |  | .473 |
|  | i32 |  |  | .466 |
|  | f19 |  |  | .443 |
|  | n53 |  |  | .420 |
|  | d11 |  |  | .392 |
| **4** | e14 | **4** | **.778** | **.745; .807** |
|  | e16 |  |  | .370 |
|  | e13 |  | . |  |
|  | d12 |  |  |  |

### Round 2 Psychometric evaluation analysis

#### Stage 1 – testing for assumptions

##### Normality and identity matrices

Data were not normally distributed (W=0.967, df=264, p< .0001), violating EFA assumptions. However, the data were factorable, as they exhibited adequate sampling adequacy (Kaiser-Meyer-Olkin Measure of Sampling Adequacy = .958), and the Bartlett Test of Sphericity was statistically significant (χ2 (df=703) = 8113.50, p < 0.001).

**Quick comment:** Although the data were not normally distributed, they met the requirements for factor analysis. The sampling adequacy was significantly higher for the ZCS38 version than for the ZCS53 version.

##### Correlations

Most items correlated well with the total score (Table 1). Item H26 (I have friends that I trust) had the lowest item-total correlation (ITC), r=0.469, with item m46 (I enjoy the process of striving toward excellence in what matters) having the highest ITC, r=.662. The detailed inter-item correlations are illustrated in Table 7. Generally, there was no evidence of multicollinearity. However, some items did not correlate reasonably with the rest. (See Table 7).

**Quick comment**: Compared to the ZCS53, there were improvements in the inter-item correlations. By analysing the inter-item correlations, the following items were candidates for stepwise deletion: c07, e18, h26, h29, i34, j38, l44, m48, m49, and m50. Item h26 was dropped from the remaining analysis because it had the lowest ITC and correlated poorly with the other items.

Table 2: Item-total correlations

| Item | Label | Item | **ITC** | Ordered threshold |
| --- | --- | --- | --- | --- |
| a01 | A01. I feel I am a valuable person even when other people don't like me. | a01 | .480^**^ |  |
| a02 | A02. I love myself just the way I am. | a02 | .508^**^ |  |
| b04 | B04. I am proud of myself. | b04 | .482^**^ | Yes |
| b05 | B05. I can do things as well as other people. | b05 | .525^**^ |  |
| b06 | B06. I feel that I'm just as worthy as other people. | b06 | .525^**^ |  |
| c07 | C07. Living with HIV has not affected my ability to do what I want in life. | c07 | .471^**^ |  |
| d10 | D010. I am confident that I can manage my treatment. | d10 | .612^**^ |  |
| d11 | D011. I try to make a way to attend my clinic appointment, even when I have some | d11 | .543^**^ |  |
| d12 | D012. I do things to try and stay healthy (e.g., eat healthy foods, drink water, | d12 | .573^**^ | Yes |
| e13 | E13. When I grow up, I will have a good family life. | e13 | .619^**^ | Yes |
| e14 | E14. When I grow up, I will have the life I want. | e14 | .602^**^ |  |
| e16 | E16. I will be able to take care of myself. | e16 | .657^**^ | Yes |
| e18 | E18. Living with HIV will not stop me from achieving my goals. | e18 | .561^**^ |  |
| f19 | F19. I am optimistic about my future. | f19 | .647^**^ | Yes |
| f21 | F21. If I feel down, I always think about what is good in my life. | f21 | .570^**^ | Yes |
| g23 | G23. I have a reason for living. | g23 | .599^**^ |  |
| h26 | H26. I have friends that I trust. | h26 | .459^**^ | Yes |
| h27 | H27. I feel like I belong in my community. | h27 | .636^**^ |  |
| h28 | H28. My family stands by me during difficult times. | h28 | .584^**^ |  |
| h29 | H29. I feel supported by the clinic/hospital staff. | h29 | .549^**^ | Yes |
| i32 | I32. I am competent and capable in the activities that are important to me. | i32 | .659^**^ |  |
| i33 | I33. I have been feeling useful. | i33 | .568^**^ |  |
| i34 | I34. I have been dealing with problems well. | i34 | .598^**^ | Yes |
| j38 | J38. I see life challenges as a chance for me to learn and grow. | j38 | .576^**^ |  |
| j39 | J39. I am confident that I can overcome the challenges in life. | j39 | .611^**^ | Yes |
| k40 | K40. When I have a problem, I think about different ways to solve the problem. | k40 | .606^**^ | Yes |
| k42 | K42. When I think of living with HIV, I remind myself that there are others who | k42 | .607^**^ |  |
| l43 | L43. I move out of my comfort zone to achieve my goal. | l43 | .629^**^ |  |
| l44 | L44. I can handle whatever comes my way. | L44 | .639^**^ | Yes |
| l45 | L45. I finish whatever I begin. | L45 | .618^**^ | Yes |
| m46 | M46. I enjoy the process of striving toward excellence in what matters. | M46 | .662^**^ | Yes |
| m47 | M47. What matters most to me in life is the contribution I make to society. | M47 | .608^**^ | Yes |
| m48 | M48. I can separate myself from my thoughts and feelings. | M48 | .571^**^ | Yes |
| m49 | M49. I have the sense that I am fully aware of what is going on around me and in | m49 | .522^**^ | Yes |
| m50 | M50. I focus on discovering the potential meaning in every situation. | M50 | .519^**^ | Yes |
| n51 | N51. I can do anything when I put my mind to it. | N51 | .661^**^ | Yes |
| n52 | N52. What happens to me in the future mostly depends on me. | N52 | .595^**^ |  |
| n53 | N53. My life is in my hands, and I am in control of it. | N53 | .609^**^ | Yes |

#### Stage 2 – Factor extraction

- Initially, factor extraction was performed using the conventional approach, with a threshold of r≥ 0.3 to omit items with low factor loadings.
- The initial solution yielded five (5) unique factors and items. However, the fifth factor consisted of one item, hence subsequent analyses were restricted to four factors for a parsimonious solution.
- No items were dropped using the conventional approach, hence the need to use an alternative approach.
- During the second iteration, the average inter-item was calculated, and the threshold for exclusion was r < 0.337. Using this approach, only item i33 was excluded, hence the need for a more stringent cut-off.
- Next, the threshold for extraction was set at r≥ 0.4; using this approach, only five items were candidates for removal.
- Afterwards, the cut-off was increased to r≥ 0.5 as this would present a moderate correlation. Unfortunately, the criteria were too stringent as only 17 items were retained against a target of 20-25 items to ensure adequate content validity.
- Subsequent analyses were conducted at the threshold r≥ 0.45.
- Table 2 (Page 4) shows outputs using maximum likelihood and generalised least square methods. In both instances, Oblimin and Promax rotations were applied to improve interpretability.

Table 3: Iterative factor extractions

| **Extraction Method: Maximum Likelihood.** | | | | | **Extraction Method: Maximum Likelihood.** | | | | **Extraction Method: Generalised Least Squares** | | | | **Extraction Method: Generalised Least Squares.** | | | |
| --- | --- | --- | --- | --- | --- | --- | --- | --- | --- | --- | --- | --- | --- | --- | --- | --- |
| Rotation Method: Promax with Kaiser Normalization. | | | |  | Rotation Method: Oblimin with Kaiser Normalization. | | | | Rotation Method: Promax with Kaiser Normalization. | | |  | Rotation Method: Oblimin with Kaiser Normalization. | | |  |
| **Item** | **Factor 1** | **Factor 2** | **Factor 3** | **Factor 4** | **Factor 1** | **Factor 2** | **Factor 3** | **Factor 4** | **Factor 1** | **Factor 2** | **Factor 3** | **Factor 4** | **Factor 1** | **Factor 2** | **Factor 3** | **Factor 4** |
| a01 |  |  |  |  |  |  |  |  |  | 0.492 |  |  |  | 0.467 |  |  |
| a02 |  | 0.645 |  |  |  | 0.618 |  |  |  | 0.565 |  |  |  | 0.585 |  |  |
| b04 |  | 0.745 |  |  |  | 0.680 |  |  |  | 0.750 |  |  |  | 0.706 |  |  |
| b05 |  |  |  |  |  |  |  |  |  |  |  |  |  |  |  |  |
| b06 |  | 0.565 |  |  |  | 0.562 |  |  |  | 0.545 |  |  |  | 0.581 |  |  |
| c07 |  | 0.451 |  |  |  |  |  |  |  |  |  |  |  | 0.458 |  |  |
| d10 |  |  |  |  |  |  |  |  |  |  |  |  |  |  |  |  |
| d11 |  |  |  |  |  |  |  |  |  |  |  |  |  |  |  |  |
| d12 |  |  |  |  |  |  |  |  |  |  |  |  |  |  |  |  |
| e13 |  |  |  |  |  |  |  |  |  |  |  | 0.505 |  |  |  |  |
| e14 |  |  |  | 0.482 |  |  |  |  |  |  |  | 0.612 |  |  | -0.451 |  |
| e16 |  |  |  |  |  |  |  | -0.456 |  |  |  | 0.461 |  |  |  | -0.498 |
| e18 |  | 0.504 |  |  |  | 0.502 |  |  |  |  |  |  |  | 0.491 |  |  |
| f19 |  |  | 0.456 |  |  |  |  | -0.461 |  |  | 0.472 |  |  |  |  | -0.466 |
| f21 |  |  |  |  | 0.464 |  |  |  |  |  |  |  | 0.453 |  |  |  |
| g23 |  |  | 0.522 |  |  |  |  | -0.489 |  |  | 0.566 |  |  |  |  | -0.520 |
| h27 |  |  |  |  | 0.463 |  |  |  | 0.457 |  |  |  | 0.477 |  |  |  |
| h28 |  |  | 0.674 |  |  |  |  | -0.619 |  |  | 0.726 |  |  |  |  | -0.654 |
| h29 |  |  | 0.555 |  |  |  |  | -0.519 |  |  | 0.591 |  |  |  |  | -0.545 |
| i32 |  |  | 0.466 |  |  |  |  |  |  |  | 0.473 |  |  |  |  |  |
| i33 |  |  |  |  |  |  |  |  |  |  |  |  |  |  |  |  |
| i34 | 0.605 |  |  |  | 0.605 |  |  |  | 0.621 |  |  |  | 0.617 |  |  |  |
| j38 |  |  | 0.508 |  |  |  |  |  |  |  | 0.515 |  |  |  |  |  |
| j39 | 0.473 |  |  |  | 0.492 |  |  |  | 0.477 |  |  |  | 0.492 |  |  |  |
| k40 | 0.458 |  |  |  | 0.474 |  |  |  | 0.489 |  |  |  | 0.495 |  |  |  |
| k42 |  |  |  |  |  |  |  |  |  |  |  |  |  |  |  |  |
| l43 | 0.638 |  |  |  | 0.639 |  |  |  | 0.652 |  |  |  | 0.646 |  |  |  |
| l44 | 0.774 |  |  |  | 0.761 |  |  |  | 0.783 |  |  |  | 0.769 |  |  |  |
| l45 | 0.596 |  |  |  | 0.604 |  |  |  | 0.602 |  |  |  | 0.611 |  |  |  |
| m46 |  |  | 0.479 |  |  |  |  |  |  |  | 0.474 |  |  |  |  |  |
| m47 | 0.637 |  |  |  | 0.636 |  |  |  | 0.641 |  |  |  | 0.644 |  |  |  |
| m48 | 0.611 |  |  |  | 0.606 |  |  |  | 0.616 |  |  |  | 0.603 |  |  |  |
| m49 | 0.533 |  |  |  | 0.536 |  |  |  | 0.539 |  |  |  | 0.540 |  |  |  |
| m50 | 0.562 |  |  |  | 0.558 |  |  |  | 0.574 |  |  |  | 0.572 |  |  |  |
| n51 |  |  |  |  |  |  |  |  |  |  |  |  |  |  |  |  |
| n52 | 0.554 |  |  |  | 0.560 |  |  |  | 0.576 |  |  |  | 0.577 |  |  |  |
| n53 |  |  |  |  |  |  |  |  |  |  |  |  |  |  |  |  |

#### Proposed Factor Solution

##### Overview

Iterative factor extraction methods are gravitating towards a 25-factor solution. The subsequent sections will report on the psychometrics of the ZCS25 factor solution obtained via the Generalised Least Squares extraction method with Promax rotation.

#### Number of factors

The Kaiser approach suggests five unique factors, i.e., factors with eigenvalues ≥1 (Table 3). However, the scree plot (Figure 1 – Page 6) supports a four-factor solution as they were an inflexion on the fifth factor. Therefore, it is essential to closely inspect the structure and pattern matrices to finalise the ZCS25 factorial structure.

Table 4: ZCS25 factor extraction

| Total Variance Explained | | | | | | | |
| --- | --- | --- | --- | --- | --- | --- | --- |
| Factor | Initial Eigenvalues | | | Extraction Sums of Squared Loadings | | | Rotation Sums of Squared Loadings^a^ |
|  | Total | % of Variance | Cumulative % | Total | % of Variance | Cumulative % | Total |
| 1 | 12.762 | 34.493 | 34.493 | 12.285 | 33.203 | 33.203 | 10.203 |
| 2 | 2.162 | 5.844 | 40.337 | 1.683 | 4.549 | 37.753 | 9.412 |
| 3 | 1.321 | 3.569 | 43.906 | .841 | 2.272 | 40.025 | 7.232 |
| 4 | 1.178 | 3.184 | 47.090 | .716 | 1.936 | 41.962 | 7.269 |
| 5 | 1.038 | 2.806 | 49.896 | .581 | 1.571 | 43.532 | 6.595 |
| 6 | .973 | 2.629 | 52.524 |  |  |  |  |
| Extraction Method: Generalised Least Squares. | | | | | | | |
| a. When factors are correlated, sums of squared loadings cannot be added to obtain a total variance. | | | | | | | |

Figure 1: scree plot

**Quick comment:** There can be as many factors as items. Given the divergent Kaiser extraction and scree plot outputs, there is a need to iteratively remove items and inspect the resultant factor matrices.

#### Stage 3 – Factor rotation and interpretation

Four factors were retained upon inspection of both structure and pattern matrices (See Table 10 Page 14). Factors underwent Promax (oblique) rotation method to improve interpretability. Further, item loading was high, ranging from 0.457 to 0.783. The structure matrix shows multiple cross-loadings for factors 1, 2 and 3. To optimise the scale to 25 items, item h27 was omitted as it has the lowest factor loading for items in factor 1. Also, item h27 had multiple cross-loadings across three factors. Outlined in Table 4 below is the proposed ZCS25 scale.

Table 5: ZCS26 matrices

| **Pattern Matrix** | | | | | **Structure Matrix** | | | |
| --- | --- | --- | --- | --- | --- | --- | --- | --- |
|  | **Factor** | | | | **Factor** | | | |
|  | **1** | **2** | **3** | **4** | **1** | **2** | **3** | **4** |
| a01 |  | 0.492 |  |  |  | 0.568 | 0.469 |  |
| a02 |  | 0.565 |  |  |  | 0.600 |  |  |
| b04 |  | 0.750 |  |  |  | 0.655 |  |  |
| b06 |  | 0.545 |  |  |  | 0.625 | 0.460 |  |
| e13 |  |  |  | 0.505 | 0.478 | 0.510 | 0.486 | 0.662 |
| e14 |  |  |  | 0.612 | 0.462 | 0.507 |  | 0.721 |
| e16 |  |  |  | 0.461 | 0.455 | 0.531 | 0.651 | 0.645 |
| f19 |  |  | 0.472 |  | 0.511 | 0.475 | 0.643 | 0.498 |
| g23 |  |  | 0.566 |  |  | 0.516 | 0.645 |  |
| h28 |  |  | 0.726 |  | 0.474 |  | 0.656 |  |
| h29 |  |  | 0.591 |  |  |  | 0.601 |  |
| i32 |  |  | 0.473 |  | 0.565 | 0.509 | 0.662 |  |
| i34 | 0.621 |  |  |  | 0.638 |  | 0.458 |  |
| j38 |  |  | 0.515 |  | 0.517 |  | 0.595 |  |
| j39 | 0.477 |  |  |  | 0.620 |  | 0.542 |  |
| k40 | 0.489 |  |  |  | 0.606 | 0.470 | 0.511 |  |
| l43 | 0.652 |  |  |  | 0.685 |  | 0.519 |  |
| l44 | 0.783 |  |  |  | 0.724 |  | 0.454 |  |
| l45 | 0.602 |  |  |  | 0.662 |  | 0.494 |  |
| m46 |  |  | 0.474 |  | 0.603 | 0.481 | 0.668 |  |
| m47 | 0.641 |  |  |  | 0.648 |  |  |  |
| m48 | 0.616 |  |  |  | 0.620 |  | 0.462 |  |
| m49 | 0.539 |  |  |  | 0.570 |  |  |  |
| m50 | 0.574 |  |  |  | 0.551 |  |  |  |
| n52 | 0.576 |  |  |  | 0.627 |  | 0.468 |  |

**Quick comment:** Items with multiple cross-loadings are candidates for removal. However, the multiple cross-loadings on the ZCS structure matrix may also imply the interconnectedness of the scale.

#### Draft ZCS25 scale with item labels

Outlined in Table 5 below is the ZCS25; items are sorted per factor.

Table 6: ZCS25 SCALE WITH LABELS

|  | **Pattern Matrix** | | | | |
| --- | --- | --- | --- | --- | --- |
|  |  | **Factor** | | | |
| **Item code** | **Description** | **1** | **2** | **3** | **4** |
| a01 | A01. I feel I am a valuable person even when other people don’t like me. |  | 0.492 |  |  |
| a02 | A02. I love myself just the way I am. |  | 0.565 |  |  |
| b04 | B04. I am proud of myself. |  | 0.750 |  |  |
| b06 | B06. I feel that I’m just as worthy as other people. |  | 0.545 |  |  |
| e13 | E13. When I grow up, I will have a good family life. |  |  |  | 0.505 |
| e14 | E14. When I grow up, I will have the life I want. |  |  |  | 0.612 |
| e16 | E16. I will be able to take care of myself. |  |  |  | 0.461 |
| f19 | F19. I am optimistic about my future. |  |  | 0.472 |  |
| g23 | G23. I have a reason for living. |  |  | 0.566 |  |
| h28 | H28. My family stands by me during difficult times. |  |  | 0.726 |  |
| h29 | H29. I feel supported by the clinic/hospital staff. |  |  | 0.591 |  |
| i32 | I32. I am competent and capable in the activities that are important to me. |  |  | 0.473 |  |
| j38 | J38. I see life challenges as a chance for me to learn and grow. |  |  | 0.515 |  |
| m46 | M46. I enjoy the process of striving toward excellence in what matters. |  |  | 0.474 |  |
| i34 | I34. I have been dealing with problems well. | 0.621 |  |  |  |
| j39 | J39. I am confident that I can overcome the challenges in life. | 0.477 |  |  |  |
| k40 | K40. When I have a problem, I think about different ways to solve the problem. | 0.489 |  |  |  |
| l43 | L43. I move out of my comfort zone to achieve my goal. | 0.652 |  |  |  |
| l44 | L44. I can handle whatever comes my way. | 0.783 |  |  |  |
| l45 | L45. I finish whatever I begin. | 0.602 |  |  |  |
| m47 | M47. What matters most to me in life is the contribution I make to society. | 0.641 |  |  |  |
| m48 | M48. I can separate myself from my thoughts and feelings. | 0.616 |  |  |  |
| m49 | M49. I have the sense that I am fully aware of what is going on around me and in | 0.539 |  |  |  |
| m50 | M50. I focus on discovering the potential meaning in every situation. | 0.574 |  |  |  |
| n52 | N52. What happens to me in the future mostly depends on me. | 0.576 |  |  |  |

#### Naming of factors:

- Factor 4 is more distinct than the other factors; it concerns hope for the future and could be abbreviated to hope.
- Factors 1-3 contain more than one construct; the items can be further disaggregated into two or more subdomains subject to the interpretation of the rest of the team. For example, for factor 1, I think items *“A01. I feel I am a valuable person even when other people don’t like me.”* and *“A02. I love myself just the way I am.”* may imply **self-acceptance** with items *“B04. I am proud of myself.”* And *“B06. I feel that I’m just as worthy as other people.”* Implying **self-worthy**; again, this is up for discussion.

#### Reliability analysis

#### Overall scale reliability

The ZCS25 yielded a high internal consistency of α= .924 and ICC= 924 (95% CI: .914: .933). Deleting any items did not improve α; this shows high item reliability.

Table 7: ZCS scale internal consistency

|  | Scale Mean if Item Deleted | Scale Variance if Item Deleted | Corrected Item-Total Correlation | Cronbach’s Alpha if Item Deleted |
| --- | --- | --- | --- | --- |
| a01 | 83.44 | 66.950 | .424 | .923 |
| a02 | 83.47 | 66.703 | .455 | .922 |
| b04 | 83.51 | 66.466 | .427 | .923 |
| b06 | 83.47 | 66.677 | .469 | .922 |
| e13 | 83.48 | 65.936 | .578 | .920 |
| e14 | 83.55 | 65.897 | .550 | .921 |
| e16 | 83.50 | 65.827 | .601 | .920 |
| f19 | 83.49 | 65.721 | .617 | .920 |
| g23 | 83.55 | 65.885 | .555 | .921 |
| h28 | 83.55 | 65.368 | .532 | .921 |
| h29 | 83.48 | 66.140 | .517 | .921 |
| i32 | 83.58 | 65.344 | .614 | .920 |
| j38 | 83.62 | 65.506 | .539 | .921 |
| m46 | 83.53 | 65.289 | .643 | .919 |
| i34 | 83.79 | 64.676 | .577 | .920 |
| j39 | 83.65 | 65.002 | .597 | .920 |
| k40 | 83.72 | 65.048 | .576 | .920 |
| l43 | 83.69 | 64.830 | .603 | .920 |
| l44 | 83.73 | 64.533 | .617 | .919 |
| l45 | 83.73 | 64.987 | .596 | .920 |
| m47 | 83.68 | 65.169 | .580 | .920 |
| m48 | 83.77 | 64.969 | .547 | .921 |
| m49 | 83.69 | 65.936 | .502 | .921 |
| m50 | 83.82 | 65.847 | .473 | .922 |
| n52 | 83.62 | 65.461 | .557 | .921 |

##### Sub-scales/domain’s reliability

ZCS25's four subscales exhibited high internal consistency, ranging from .707 to .877; this is evidence of high subscale reliability (Table 7). Subscale reliability is a function of the number of items, i.e., the greater the number of items, the higher the α.

Table 8: ZCS SUB-SACLES INTERNAL CONSISTENCY

|  |  | **# of items** | **α** | **ICC (95% CI)** |
| --- | --- | --- | --- | --- |
|  | **Description** |  |  |  |
| **Factor 1** | A01. I feel I am a valuable person even when other people don’t like me. | 4 | .707 | .707 (.664: .746) |
|  | A02. I love myself just the way I am. |  |  |  |
|  | B04. I am proud of myself. |  |  |  |
|  | B06. I feel that I’m just as worthy as other people. |  |  |  |
| **Factor 2** | E13. When I grow up, I will have a good family life. | 3 | .752 | .752 (.713: .786) |
|  | E14. When I grow up, I will have the life I want. |  |  |  |
|  | E16. I will be able to take care of myself. |  |  |  |
| **Factor 3** | F19. I am optimistic about my future. | 7 | .825 | .825 (.801: .847) |
|  | G23. I have a reason for living. |  |  |  |
|  | H28. My family stands by me during difficult times. |  |  |  |
|  | H29. I feel supported by the clinic/hospital staff. |  |  |  |
|  | I32. I am competent and capable in the activities that are important to me. |  |  |  |
|  | J38. I see life challenges as a chance for me to learn and grow. |  |  |  |
|  | M46. I enjoy the process of striving toward excellence in what matters. |  |  |  |
| **Factor 4** | I34. I have been dealing with problems well. | 11 | .877 | .877 (.861: .892) |
|  | J39. I am confident that I can overcome the challenges in life. |  |  |  |
|  | K40. When I have a problem, I think about different ways to solve the problem. |  |  |  |
|  | L43. I move out of my comfort zone to achieve my goal. |  |  |  |
|  | L44. I can handle whatever comes my way. |  |  |  |
|  | L45. I finish whatever I begin. |  |  |  |
|  | M47. What matters most to me in life is the contribution I make to society. |  |  |  |
|  | M48. I can separate myself from my thoughts and feelings. |  |  |  |
|  | M49. I have the sense that I am fully aware of what is going on around me and in |  |  |  |
|  | M50. I focus on discovering the potential meaning in every situation. |  |  |  |
|  | N52. What happens to me in the future mostly depends on me. |  |  |  |

#### Scoring instructions

- There has been consensus to apply a four-point Likert scoring system and preferably to use the same scoring options throughout.
- Table 8 below shows potential scaling options.

Table 9: potential scoring options

| **Response classification** | **Potential phrasing of the overall scoring options** | **Options** |
| --- | --- | --- |
| Agreement | To what extent do you agree with the following statements… | 4- strongly agree  3-Agree  2- disagree  1- strongly disagree |
| Frequency | In the past _ days/weeks, how frequent were you feeling or experiencing the following feelings/emotions … | 4- Most of the time  3- Some of the time  2- seldom  1- never |
|  |  | 4- Almost always  3- Often  2- seldom  1- never |
|  |  | 4- A great deal  3- Occasionally  2- Rarely  1-Never |

##### CS25 Scale Version 1.0

Table 1 below shows the first version of the ZCS25 and corresponding factors/items. The rest of the document outlines the item reduction and reliability testing processes.

Table 1: ZCS25

|  |  | **# of items** |
| --- | --- | --- |
|  | **Description** |  |
| **Factor 1** | A01. I feel I am a valuable person even when other people do not like me. | 4 |
|  | A02. I love myself just the way I am. |  |
|  | B04. I am proud of myself. |  |
|  | B06. I feel that I’m just as worthy as other people. |  |
| **Factor 2** | E13. When I grow up, I will have a good family life. | 3 |
|  | E14. When I grow up, I will have the life I want. |  |
|  | E16. I will be able to take care of myself. |  |
| **Factor 3** | F19. I am optimistic about my future. | 7 |
|  | G23. I have a reason for living. |  |
|  | H28. My family stands by me during difficult times. |  |
|  | H29. I feel supported by the clinic/hospital staff. |  |
|  | I32. I am competent and capable in the activities that are important to me. |  |
|  | J38. I see life challenges as a chance for me to learn and grow. |  |
|  | M46. I enjoy the process of striving toward excellence in what matters. |  |
| **Factor 4** | I34. I have been dealing with problems well. | 11 |
|  | J39. I am confident that I can overcome the challenges in life. |  |
|  | K40. When I have a problem, I think about different ways to solve the problem. |  |
|  | L43. I move out of my comfort zone to achieve my goal. |  |
|  | L44. I can handle whatever comes my way. |  |
|  | L45. I finish whatever I begin. |  |
|  | M47. What matters most to me in life is the contribution I make to society. |  |
|  | M48. I can separate myself from my thoughts and feelings. |  |
|  | M49. I have the sense that I am fully aware of what is going on around me and in |  |
|  | M50. I focus on discovering the potential meaning in every situation. |  |
|  | N52. What happens to me in the future mostly depends on me. |  |
